# Human Amniotic Mesenchymal Stem Cells Promote Endogenous Bone Regeneration

**DOI:** 10.3389/fendo.2020.543623

**Published:** 2020-10-02

**Authors:** Jin Li, Zhixuan Zhou, Jin Wen, Fei Jiang, Yang Xia

**Affiliations:** ^1^Jiangsu Key Laboratory of Oral Diseases, Nanjing Medical University, Nanjing, China; ^2^Department of General Dentistry, Affiliated Hospital of Stomatology, Nanjing Medical University, Nanjing, China; ^3^Department of Prosthodontics, School of Medicine, College of Stomatology, Shanghai Ninth People's Hospital, Shanghai Jiao Tong University, Shanghai, China; ^4^Department of Prosthodontics, Affiliated Hospital of Stomatology, Nanjing Medical University, Nanjing, China

**Keywords:** hAMSCs, paracrine functions, endogenous bone regeneration, arthritis, bone defects, clinical trials

## Abstract

Bone regeneration has become a research hotspot and therapeutic target in the field of bone and joint medicine. Stem cell-based therapy aims to promote endogenous regeneration and improves therapeutic effects and side-effects of traditional reconstruction of significant bone defects and disorders. Human amniotic mesenchymal stem cells (hAMSCs) are seed cells with superior paracrine functions on immune-regulation, anti-inflammation, and vascularized tissue regeneration. The present review summarized the source and characteristics of hAMSCs and analyzed their roles in tissue regeneration. Next, the therapeutic effects and mechanisms of hAMSCs in promoting bone regeneration of joint diseases and bone defects. Finally, the clinical application of hAMSCs from current clinical trials was analyzed. Although more studies are needed to confirm that hAMSC-based therapy to treat bone diseases, the clinical application prospect of the approach is worth investigating.

## Introduction

Large bone defects and disorders, either congenital or acquired, severely affect the patients' appearance and function (1). Moreover, the incidence of these bone diseases is high and has been on the rise in the past decade. In terms of bone defects, approximately more than 350 million people suffer from fractures, about 46 million people have head injuries, and 20 million people are subject to spinal injuries. Meanwhile, more than 300 million people have been diagnosed with osteoarthritis while about 20 million people have got rheumatoid arthritis around the world. Periodontal disease, which tends to cause severe alveolar bone loss and tooth loss, also has a high prevalence rate of about 800 million (2). Currently, the reconstruction of the bone defects mainly depends on autologous tissue transplantation due to various factors, such as biocompatibility and histocompatibility (3). However, this strategy has limited applications due to a shortage of harvest sites, incomplete integration into the defects, and risk of disease transfer (4). In addition to various defects, bone disorders also include joint diseases associated with an autoimmune disorder, such as rheumatoid arthritis (RA) (5), osteoarthritis (OA) (6), and ankylosing spondylitis (AS) (7). Typically, these joint diseases are treated by drugs (for example, glucocorticoid, immunosuppressive agents, non-steroidal anti-inflammatory drugs, and disease modifying antirheumatic drug) to reduce the symptoms and improve the joint function; however, therapeutic interventions are essential in the advanced stage characterized by loss of articular cartilage, subchondral sclerosis, osteophyte formation, and joint capsule thickening (8). Nowadays, tissue engineering is gaining increasing attention and is expected to resolve these clinical issues.

Different types of scaffold, bioactive factors, and seed cells are the three major elements of tissue engineering. Thus, finding an ideal scaffold to replace the autologous bone translation in the treatment of bone disorder is under intensive research. Nowadays, the scaffold matrix used for bone tissue engineering includes inorganic material, polymers, and their composites (9). In addition, bioactive factors, such as bone morphogenetic protein-2 (BMP-2), fibroblast growth factor-2 (FGF-2), vascular endothelial growth factor (VEGF), and epidermal growth factor (EGF), also play a vital role in bone rebuilding (10). Among those growth factors, BMP-2 and FGF-2 have been utilized to promote bone regeneration and angiogenesis in clinical practice (11, 12). Currently, stem cell-based tissue regeneration has some curative effects (13), but the effect of seed cells in repairing the bone disorders is yet controversial.

Endogenous regeneration, proposed in recent years, focuses on the stimulation and regulation of endogenous factors to achieve *in situ* tissue regeneration by applying bioactive factors locally (14). Stem cells possess robust biological potential with respect to self-renewal, multidirectional differentiation, and paracrine functions (15, 16). These might act as bioactive factors to activate the endogenous regeneration by local or systematic applications (14, 17). The homeostasis of tissues and organs relies on the coordination and regulation of the nervous, endocrine, and immune systems (18). The endocrine system is a complex network of hormone-producing cells and tissues, which secrete a variety of hormones to act on distant and/or adjacent target cells through endocrine, paracrine, autocrine, or intracrine mechanisms to exert biological activities (19). In addition to enteroendocrine cells, other tissues and cells, such as retinal ganglion cells (RGCs) (20), bone (21), and muscle (22), have paracrine and endocrine functions to maintain homeostasis. Growth hormone (GH) can be expressed in RGCs, and retinal GH has a paracrine role in ocular development and vision (20). It has been widely accepted that stem cells could secrete a variety of bioactive factors which regulate immune state of the body and local microenvironment of tissue regeneration (23). These mechanisms of stem cell-based therapy are to some extent similar to those of some hormones, such as GH. Based on these similarities, stem cell-based therapy might exert a positive effect on promoting endogenous bone regeneration.

Stem cells are divided into embryonic stem cells and adult stem cells (16). The embryonic stem cells for stem cell therapy shows high tumorigenicity and ethical problems in the application process (24). Adult stem cells, including mesenchymal stem cells (MSCs), are undifferentiated cells found in various tissues and organs (25). Nowadays, researchers can isolate MSCs from bone marrow (bone-marrow mesenchymal stem cells, BMSCs) (26), fat (adipose-derived stem cells, AdSCs) (27), peripheral blood (peripheral blood-derived mesenchymal stem cells, PMSCs) (28), umbilical cord blood (umbilical cord blood-derived mesenchymal stem cells, CB-MSCs) (29), and other tissues (30–32) for tissue engineering, immune-regulation, and anti-inflammation. However, it is also unknown which source of stem cells is better for promoting tissue regeneration after transplantation.

Currently, we are focusing on promoting bone regeneration in the oral and maxillofacial regions using human amniotic mesenchymal stem cells (hAMSCs). In this study, we reviewed the source, characteristics, and roles of hAMSCs in bone regeneration, not only in the reconstruction of bone defects but also in the treatment of arthritis. Thus, hAMSCs might be used as an innovative treatment option to promote endogenous bone regeneration.

## Source and Characteristics of HAMSCs

MSCs are specialized cells with multi-differentiation potentials, which can be activated to differentiate into tissue cells under specific inducing conditions (33, 34). Previous studies have demonstrated that MSCs have abilities of regeneration and immunoregulation (35). The hAMSCs, isolated from the amniotic membrane (AM) of the human term placenta that plays a key role in maintaining maternal-neonatal tolerance, not only share phenotypes similar to typical MSCs, including fibroblast-like morphology, specific surface molecules, and multi-differentiation potential but also have superior immunomodulatory (36–39) and paracrine properties (40, 41). Compared to hAMSCs, most MSCs have inevitable disadvantages on clinical use, including invasive access procedure, host immune response after transplantation, age-related heterogeneity in the quality of MSCs, and extremely low acquisition rate of MSCs (33).

The AM is the innermost layer of the placenta consisting of two sets of cells; one is the amnion epithelial cells that are in direct contact with the amniotic fluid, and the other is the amnion MSCs dispersed in the matrix (42, 43). Since AM is an avascular structure and its epithelial layer can be easily removed by Dispase II, the hAMSCs can be obtained without contamination of endothelial cells and hematopoietic cells (42, 44). Each gram of wet amnion tissue can provide 1.7 ± 0.3 × 10^6^ hAMSCs (45), which are positive for CD44 and CD90 (46, 47). Moreover, the placental tissue becomes a medical waste after childbirth, and hAMSCs can be harvested non-invasively and without ethical controversy (48). In addition, parturients are usually young women, and hence, age-related heterogeneity of hAMSCs might be relatively better than that of stem cells from other sources. The hAMSCs lack the expression of human major histocompatibility complex (MHC) antigens (human leukocyte antigens, HLA), including HLA class I antigens (HLA-DP, HLA-DA, HLA-DR) and HLA class I antigens (HLA-A, HLA-B, HLA-C), showing low immunogenicity (49, 50), while they also exhibit low tumorigenicity due to lack of expression of telomerase (48, 51, 52). Low immunogenicity and low tumorigenicity of hAMSCs render them conducive for allotransplantation to promote tissue regeneration. Also, their paracrine properties have multiple regulatory functions (40). Furthermore, several bioactive factors could be produced by hAMSCs, including immunomodulatory factors that are crucial for the resolution of inflammation (44, 53) and growth and angiogenic factors that are critical for tissue remodeling (41). These exogenous molecules have been shown to be important in inducing endogenous regeneration.

## Efficacy of HAMSCs in Joint Diseases

Arthritis is a characteristic of rheumatic diseases, which are chronic, intractable, and musculoskeletal system diseases, such as RA, OA, AS, and juvenile idiopathic arthritis (JIA) (54). Although the pathological characteristics of these joint disorders are different, the joint symptoms are associated with abnormal autoimmune function, inflammatory cell infiltration, and joint structural lesions (5). Stem cells, including hAMSCs, have been introduced to arthritis models, such as rat, to improve the treatment by inhibiting inflammation, regulating the status of autoimmunity, and promoting tissue regeneration (Table 1) (55, 64, 65).

**Table 1:** The mechanisms of hAMSCs in regulating joint diseases.

**References**	**Disease Model**	**Method**	**Conclusion**
Shu et al. (55)	RA	Intraperitoneal injection	hAMSCs inhibited the production of proinflammatory cytokines and the response of T-cell, and restored CD4+/CD8+ T cell ratio in CIA rats.
Parolini et al. (56)	RA	Subcutaneous injection	hAMSCs decreased the production of inflammatory cytokines, stimulated the generation of human CD4+CD25+FoxP3+ Treg cells, and suppressed the antigen-specific Th1/Th17 activation in CIA mice.
Huss et al. (57)	OA	Culture *in vitro*	NK cells were a principal infiltrating immune cells in synovial tissue of patients with osteoarthritis.
Pianta et.al. (58)	Inflammation	Co-culture	hAMSC-CM regulated T-cell polarization toward Th1, Th2, Th17, and T-regulatory (Treg) subsets.
Topoluk et al. (59)	OA	Co-culture	hAMSCs were better than AdSCs in shifting OA synovial macrophage M1:M2 ratio.
Cargnoni et al. (60)	Lung fibrosis	Intrathoracic injection	hAMSC-CM reduced the levels of pro-inflammatory and pro-fibrotic cytokines, and reduced lung macrophage levels.
Borem et al. (61)	IVDD	Co-culture	hAMSCs produced more anti-inflammatory cytokines than AdSCs under identical inflammatory conditions.
Miceli et al. (62)	Inflammation	Culture *in vitro*	hAMSCs in 3D culture system produced more angiogenic and immunosuppressive factors than in 2D cultures.
Banerjee et al. (63)	Inflammation	Culture *in vitro*	hAMSCs changed mitochondrial function and increased IL-6, and maintained the low levels of ROS at 20% oxygen.

*RA, rheumatoid arthritis; hAMSCs, human amniotic mesenchymal stem cells; CIA, collagen-induced arthritis; OA, osteoarthritis; hAMSC-CM, hAMSCs conditioned medium; AdSCs, human adipose stem cells; IVDD, intervertebral disc degeneration; 3D, three-dimensional; 2D, two-dimensional; ROS, reactive oxygen species*.

RA is a chronic, autoimmune, inflammatory joint disease characterized by hyperplasia of the synovial membrane and infiltration of immune and inflammatory cells. The synovial cell fibrosis, excessive production of inflammatory cytokines, and osteoclast appearance led to joint destruction and disability (5). The immunomodulatory and anti-inflammatory properties of hAMSCs indicated the therapeutic potential for the treatment of RA. In the classic rat arthritis model for human RA, hAMSCs significantly ameliorated the severity of arthritis and decreased the histopathological changes due to dramatic inhibition of the production of proinflammatory cytokines, such as interferon-γ (IFN-γ) and tumor necrosis factor-α (TNF-α) (55). For a T cell-mediated disease, such as RA, the therapeutic effects of hAMSCs are crucial because they could remarkably restore the CD4+/CD8+ T-cell ratio and inhibit the response of T-cells (55). In addition, hAMSCs suppressed the antigen-specific Th1/Th17 activation and stimulated the generation of CD4+CD25+FoxP3+ Treg cells (56). In mice with collagen-induced arthritis (CIA), systemic infusion of hAMSCs markedly reduces Th1-driven autoimmunity and inflammation, as shown by decreased production of TNF-α, IFN-γ, and some interleukins (IL-2 and IL-17) and increased production of IL-10 and activation of cyclooxygenase 1/2 (COX1/2) (56).

OA is another chronic joint disease with an incidence as high as 40% (66). It is a degenerative disease characterized by progressive cartilage degradation, subchondral bone remodeling, osteophyte formation, and synovitis (67). Synovial NK cells and macrophages secrete abnormally large amounts of perforins, granzymes, and pro-inflammatory cytokines (TNF-α, IL-1β) to induce and aggravate the synovial inflammation and bone/cartilage resorption (57), while activation of CD4+Th1 cells contributes to the development of inflammation (68). The hAMSCs might be beneficial for OA as they inhibit the proliferation of T-cells *in vitro* (58), polarize M2 macrophages in the condition with the hallmarks of RA *in vitro* (59), and promote bone/cartilage regeneration in rabbits (69, 70). The conditioned medium of hAMSCs (hAMSC-CM) was reported to remedy tissue fibrosis by lowering the levels of T-cells and macrophages, leading to a decline in pro-inflammatory cytokines (60). When hAMSCs were introduced to the OA model established by coculturing the OA patients' cartilage and synovium, the M1:M2 percentage ratio of synovial macrophages was decreased significantly; also, the concentrations of IL-1β and matrix metalloproteinase-13 (MMP-13) was declined, and macrophage-mediated cartilage destruction was effectively abrogated (59).

In addition, hAMSCs not only secreted active factors with therapeutic effects routinely but also produced abundant cytokines in specific environments. Under identical inflammatory conditions, hAMSCs produce more anti-inflammatory cytokines than AdSCs, such as IL-10 (61). Under three-dimensional (3D) culture conditions, hAMSCs spheroids could secrete considerable amounts of angiogenic and immunosuppressive factors while remaining viable and multipotent (62). The production of some cytokines by hAMSCs vary under different conditions of different oxygen tension. Consequent to exposure to 20% oxygen culture condition, hAMSCs secrete abundant IL-6 as a response to changes in the mitochondrial function, but the content of intracellular reactive oxygen species (ROS) remained unaltered (63). These properties of hAMSCs rendered the stem cell-based therapy applicable and also provided valuable guidance for bone regeneration.

## Efficacy of HAMSCs in Bone Defects

The hAMSCs, as a kind of stem cells, can be induced to osteogenic differentiation to form refracted crystal-like nodules which could be indicated by alkaline phosphatase staining or/and alizarin red S staining (71–73). The expression of osteogenesis-related genes and proteins, such as alkaline phosphatase (ALP), runt-related transcription factor 2 (Runx2), osteopontin (OPN), and osteocalcin (OCN), was significantly enhanced in osteo-induced hAMSCs (74–76); also, their osteogenic differentiation capacity is superior to other MSCs derived from chorionic membrane and decidua (77, 78). However, the comparison of the osteogenic capacity of hAMSCs and BMSCs revealed that BMSCs are more likely to differentiate into osteoblasts *in vitro* and seem to be appropriate for bone regeneration (79). Nevertheless, the capacity of hAMSCs might not be inferior to that of BMSCs in improving bone regeneration *in vivo*. Several studies have reported that transplanted MSCs might play therapeutic roles by paracrine signaling rather than becoming target tissue cells directly (80–82). In recent years, several studies have focused on hAMSCs promoting tissue regeneration, based on their functions of anti-inflammation, pro-angiogenesis, and chemotaxis(Table 2) (38, 83, 96). To the best of our knowledge, hAMSCs secrete more cytokines than BMSCs, including interleukins (IL-6 and IL-8), C-X-C motif chemokine ligand-1/5 (CXCL1 and CXCL5), Angiogenin (ANG), hepatocyte growth factor (HGF), and fibroblast growth factor-7 (FGF-7) (83). These paracrine properties of hAMSCs make them suitable for the restoration of bone defects (40, 83).

**Table 2:** The role of hAMSCs in bone regeneration.

**References**	**Disease model**	**Method**	**Conclusion**
Yin et al. (69)	MSFE	Intravenous injection	hAMSCs accelerated mineralized deposition rates and enhanced bone regeneration after MSFE.
Topoluk et al. (71)	Bone defects	Culture *in vitro*	hAMSCs had a greater differentiation potential toward bone and cartilage compared with AdSCs.
Li et al. (72)	Bone defects	Implantation with PLGA	The BMP9-induced osteogenic differentiation and angiogenesis of hAMSCs could be inhibited by Schnurri-3.
Li et al. (73)	Bone defects	Implantation with scaffolds	The osteogenic differentiation and angiogenesis of hAMSCs could be enhanced by 3D silk fibroin scaffolds.
Leyva-Leyva et al. (75)	Bone defects	Culture *in vitro*	Different hAMSCs subpopulations had dissimilar osteoblastic differentiation potential, and CD105– cells were better than CD105+ cells.
Fan et al. (76)	Bone defects	culture *in vitro*	<1.0 mM sodium butyrate enhanced the expression of osteogenesis-related genes and proteins of hAMSCs.
Shen et al. (77)	Bone defects	Culture *in vitro*	hAMSCs and UC-MSC had a higher osteogenic differentiation potential than the MSCs from chorionic membrane and decidua.
Ma et al. (78)	Bone defects	Culture *in vitro*	hAMSCs had a greater osteogenic differentiation than the MSCs from umbilical cord and chorionic plate.
Liu et al. (80)	Osteopenia	Hypodermic implantation	MSCs secreted exosomes to regulate the miR-29b/Dnmt1/Notch epigenetic cascade.
Jiang et al. (83)	Bone defects	Subcutaneous injection	hAMSCs stimulated endogenous regeneration of bone via paracrine function.
Zhang et al. (84)	Osteoporosis	Co-culture	hAMSCs enhanced the cell proliferation, antioxidant properties, osteogenic, and angiogenic differentiation of BMSCs and HUVECs.
Wang et al. (85)	Periodontitis	Culture *in vitro*	hAMSCs promoted the osteoblastic differentiation of BMSCs and influenced p38 MAPK signaling to reducing bone loss.
Wang et al. (86)	Bone defects	Co-culture	hAMSCs regulated the differentiation processes in BMSCs by influencing the differentiation antagonizing non-protein coding RNA.
Zhang et al. (87)	Bone defects	Co-culture	hAMSCs increased the proliferation and osteoblastic differentiation of AdSCs and enhanced angiogenic potential of AdSCs via secretion of VEGF.
Wang et al. (88)	Bone defects	Culture *in vitro*	hAMSCs enhanced the osteogenesis of AdSCs by promoting APN excretion through APPL1-ERK1/2 activation.
Ma et al. (89)	Bone volume inadequacy	Hypodermic implantation	hAMSCs promoted osteogenic differentiation of BMSCs via H19/miR-675/APC pathway.
Wang et al. (90)	Bone defects	Co-culture	hAMSCs promoted BMSCs proliferation and osteogenic differentiation *in vitro*.
Wang et al. (91)	Bone deficiency	Culture *in vitro*	hAMSCs promoted the proliferation and osteoblastic differentiation of BMSCs via ERK1/2 MAPK signaling, and down-regulated ROS level.
Bian et al. (92)	Bone deficiency	Co-culture	hAMSCs/BMSCs cultured in transwell coculture system had better performance in bone regeneration than those in mixed coculture systems.
Yuan et al. (93)	Bone defects	Co-culture	hAMSCs promoted angiogenesis regulating by the expression of lncRNA H19.
Ranzoni et al. (94)	OI	Intraperitoneal injection	hAMSCs accelerated the bone formation via differentiating into osteoblasts and promoting endogenous osteogenesis and the maturation of resident osteoblasts.
Tsuno et al. (95)	Bone defects	Implantation with scaffolds	hAMSCs promoted bone regeneration via increasing ALP activity, calcium deposition, and the expression of osteocalcin mRNA.

*MSFE, maxillary sinus floor elevation; hAMSCs, human amniotic mesenchymal stem cells; AdSCs, adipose-derived stem cells; BMP9, bone morphogenetic protein 9; PLGA, poly(lactic-co-glycolic acid); 3D, three-dimensional; UC-MSC, umbilical cord mesenchymal stem cells; MSCs, mesenchymal stem cells; BMSCs, bone marrow mesenchymal stem cell; HUVECs, human umbilical vein endothelial cells; MAPK, mitogen-activated protein kinase; VEGF, vascular endothelial growth factor; APN, adiponectin; APPL1, adaptor protein; PH, phosphotyrosine interaction, domain and leucine zipper containing 1; ERK1/2, extracellular signaling-regulated kinase 1/2; APC, adenomatous polyposis coli; ROS, reactive oxygen species; OI, osteogenesis imperfecta; ALP, alkaline phosphatase*.

Fracture healing is a complex, well-orchestrated, regenerative process that is coordinated by a variety of cell types, including inflammatory cells, vascular endothelial cells, MSCs, and fibroblasts (97). In the various stages of the bone healing process, inflammation and angiogenesis precede osteogenesis, thereby indicating that controlling inflammation and promoting angiogenesis in an early stage might speed up the subsequent bone formation and ultimately bone remodeling (97). Therefore, we proposed to introduce hAMSCs to bone defects' microenvironment and stimulate and accelerate the endogenous vascularized bone regeneration. Several studies have shown that hAMSCs enhance the osteogenic differentiation of AdSCs, BMSCs, and promote the tube-formation of human umbilical vein endothelial cells (HUVECs) (84–89). When hAMSCs are co-cultured with BMSCs in a transwell system, ALP activity of BMSCs and the expression of osteogenic markers, including OCN and Runx2, were upregulated (90). Conversely, in the co-culture, hAMSCs reverse the inhibition of oxidative stress-induced osteogenic differentiation of caused by hydrogen peroxide, which in turn, inhibits the inflammatory response *in vivo* (91). When hAMSCs are co-cultured with HUVECs, high levels of Collagen-1 (COL1), ANG, and VEGF were detected in the co-culture medium, and capillary-like tube structures were observed in HUVEC tube-formation assay (92). Interestingly, a correlation was established between the high expression of lncRNA H19 and the pro-angiogenic functions of hAMSCs (93).

Based on the *in vitro* data, the researchers applied hAMSCs to animal bone defect models. Ranzoni et al. injected hAMSCs intraperitoneally into mice that suffered from osteogenesis imperfecta (OI). The transplanted mice had improved bone structural quality, high mineral density, and better mineralization, while the genes related to osteogenesis were upregulated and those associated with inflammation, TGF-β, and osteoclast differentiation were downregulated (94). The β-tricalcium phosphate (TCP) scaffolds containing xenograft hAMSCs have been reported to improve regeneration of Wistar rats' skull defects. The xenograft cells did not cause obvious immune response in the transplanted rats (95). In our recent studies, we comprehensively analyzed the survival of hAMSCs after transplantation in nude mice and the specific mechanism of hAMSCs in promoting bone tissue regeneration. It had been confirmed that hAMSCs could be survival in bone defects for at least 2 weeks after transplantation. Although hAMSCs survived *in vivo*, they did not seem to transform into osteoblasts. In specimens from early bone defect healing, hAMSCs polarized macrophages to M2 that could secrete pro-angiogenic and osteogenic cytokines, such as BMP-2 and VEGF (83). Moreover, we also found that hAMSCs promote extracellular matrix remodeling (98). Combined with these functions, we believed hAMSCs could start endogenous vascularized bone regeneration. And in terms of the ultimate osteogenic effect, our findings showed that hAMSCs accelerated new bone formation not only in bone defects but promoted rapid osseointegration of dental implants (69, 83).

## Clinical Trials

Stem cell therapies exert their effects on a wide range of diseases and injuries, including immune disorders (99), various neural disorders or injuries (100), myocardial injury (101), pulmonary diseases (102), diabetes (103), cancer treatments (104), and bone/cartilage degenerative disorders or injuries (105). Several types of stem cells have been subjected to clinical trials (106). Stem cells derived from the human placenta have been reported to be in clinical trials for a variety of therapeutic applications (107). In a single-center, non-randomized, intravenous dose-escalation phase Ib trial, patients with idiopathic pulmonary fibrosis received intravenous administration of placental MSCs from unrelated donors. Previous data demonstrated that placental MSC therapy is feasible and has a satisfactory short-term safety profile in idiopathic pulmonary fibrosis (108). Clinical trials using placental MSCs to treat OA, Crohn's disease, and multiple sclerosis (MS) have shown that the cells were well-tolerated, and cell therapy was dose-related (109–111). Amnion is a part of the placenta that has been used in clinical trials to treat skin and corneal burns (112, 113). These clinical trials suggested that the amniotic membrane accelerates recovery via inhibiting inflammation and releasing growth factors. These therapeutic effects could also be found in hAMSC-CM. In the subsequent clinical trials using this conditioned media, the bioactive cytokines, such as VEGF, FGFs, and keratinocyte growth factor (KGF) were identified and were found to promote ulcer healing (114) and improved photoaging (115) when the medium was locally injected into the lesions. Currently, there are no reports on the clinical trials using hAMSCs on bone regeneration; however, BMSCs (116, 117), AdSCs (118), and dental pulp stem cells (DPSCs) (119) had shown to be safe, feasible, and effective (120). Based on the data of the clinical trials, we speculated that hAMSCs could be studied in the clinical trials of bone regeneration for future applications.

## Conclusion and Perspectives

Although hAMSCs have become an alternative source of stem cells in regenerative medicine and tissue engineering due to their advantages such as easy gain, sufficient quantity, and superior properties, the application from laboratory research to clinical practice in the future needs further exploration. We still need to carry out further studies on hAMSCs acquisition, storage, and transportation to form standardized standards to maintain and improve the therapeutic potential of hAMSCs, so as to ensure the clinical application effects. It has been well-known that autologous MSCs represent the primary sources considered safe for transplantation and minimization of immunological risk. Preclinical studies should confirm the safety and immunological risk of allogenic hAMSCs for transplantation by comparing with autologous MSCs, and then the mechanisms of hAMSCs in promoting skeletal system diseases *in vivo* are also needed to further elucidate, which are important to determine the indications, timing, dosage, and accurate administration of hAMSC-based therapy. For the treatment of bone regeneration and other bone disorders, more efforts should be made to optimize the therapeutic effects of hAMSC-based therapy by combining with other biomaterials and bioactive factors. Despite these challenges, it is no doubt that MSC-based therapy has a promising clinical application prospect. Since previous studies have demonstrated the hAMSCs with excellent MSC properties, hAMSC-based therapy is worthy to be further studied in depth and finally put into clinical practice.

## Author Contributions

JL and ZZ helped to draft the manuscript. FJ and YX contributed to the conception and supervision of the study. JW contributed to the consultation and revision of the manuscript. All authors contributed to the article and approved the submitted version.

## Conflict of Interest

The authors declare that the research was conducted in the absence of any commercial or financial relationships that could be construed as a potential conflict of interest.
